# Supplementary figures and images for: Multivariate time-series analysis of biomarkers from a dengue cohort offers new approaches for diagnosis and prognosis
Source: PLoS Negl Trop Dis. 2020 Jun 16;14(6):e0008199. doi: 10.1371/journal.pntd.0008199 (PMC7380649; doi:10.1371/journal.pntd.0008199)

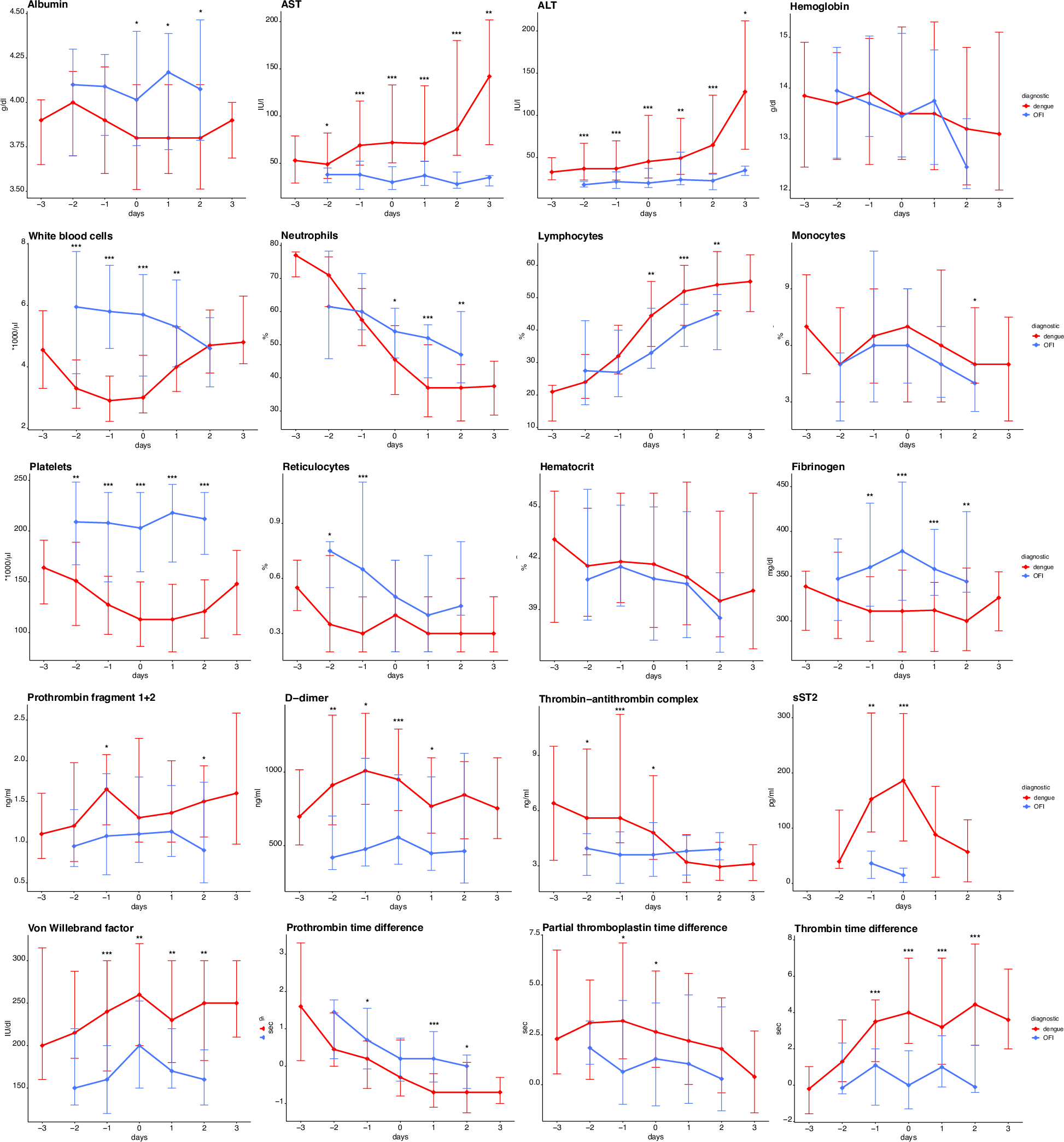

Supplement: S1 Fig — The linking lines are displayed for a better visualization but do not represent the evolution of the biomarkers at patient level. *p-value ≤ 0.05; **p-value ≤ 0.01; ***p-value ≤ 0.001. (TIF) [file pntd.0008199.s009.tif]

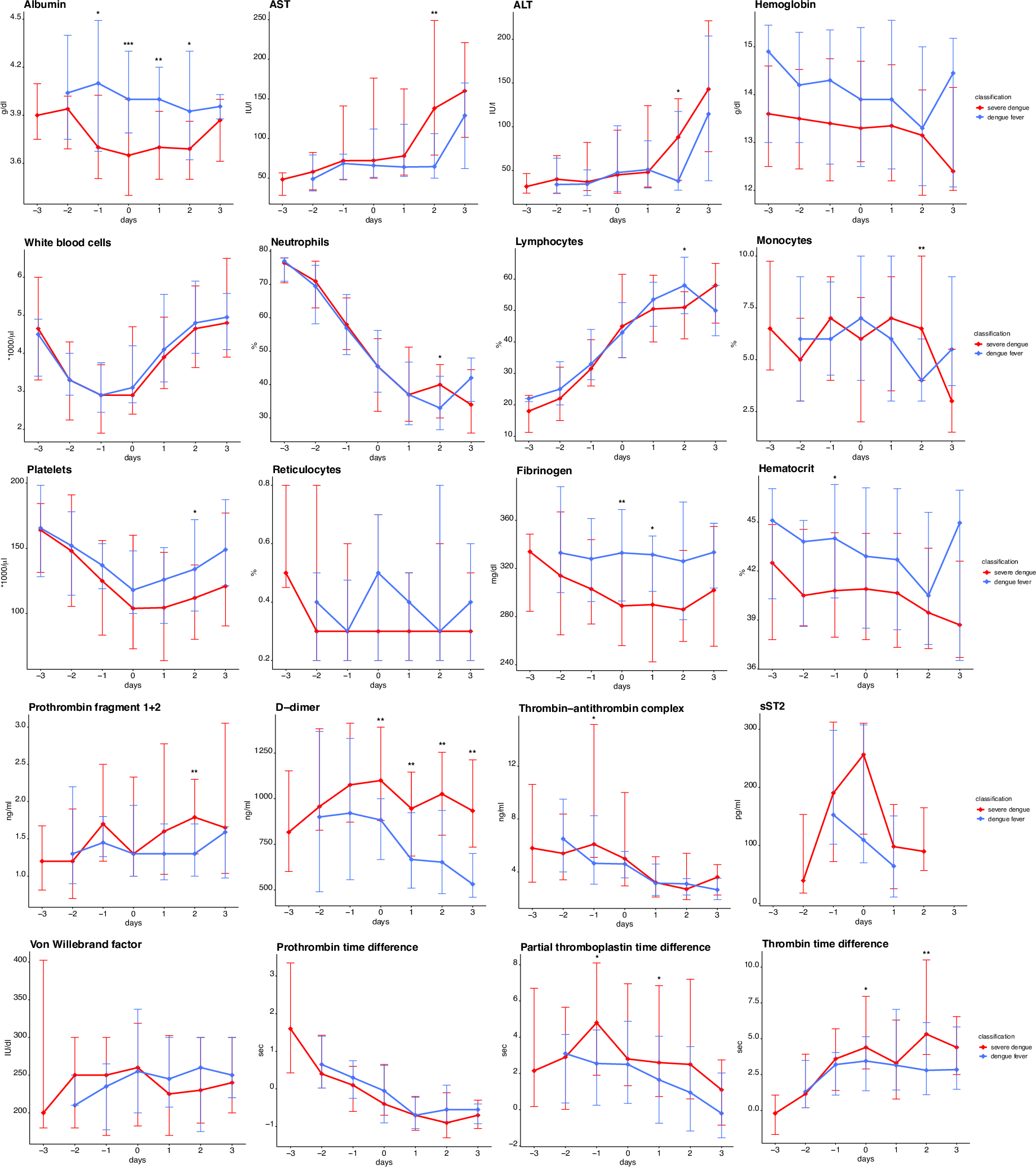

Supplement: S2 Fig — The lower and upper error bars represent the first and third quartiles respectively. The linking lines are displayed for a better visualization but do not represent the evolution of the biomarkers at patient level. *p-value ≤ 0.05; **p-value ≤ 0.01; ***p-value ≤ 0.001. (TIF) [file pntd.0008199.s010.tif]

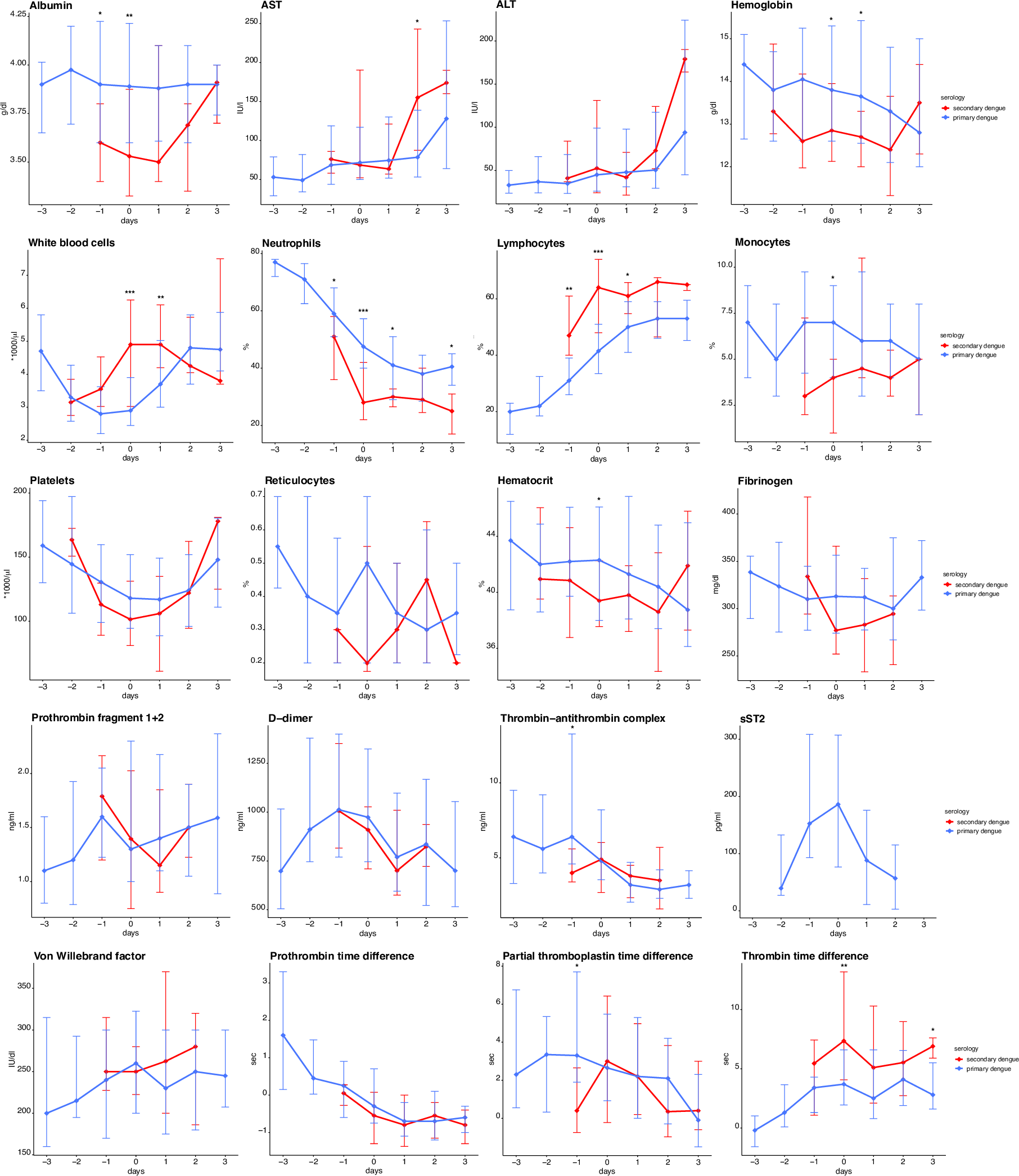

Supplement: S3 Fig — The lower and upper error bars represent the first and third quartiles respectively. The linking lines are displayed for a better visualization but do not represent the evolution of the biomarkers at patient level. *p-value ≤ 0.05; **p-value ≤ 0.01; ***p-value ≤ 0.001. (TIF) [file pntd.0008199.s011.tif]

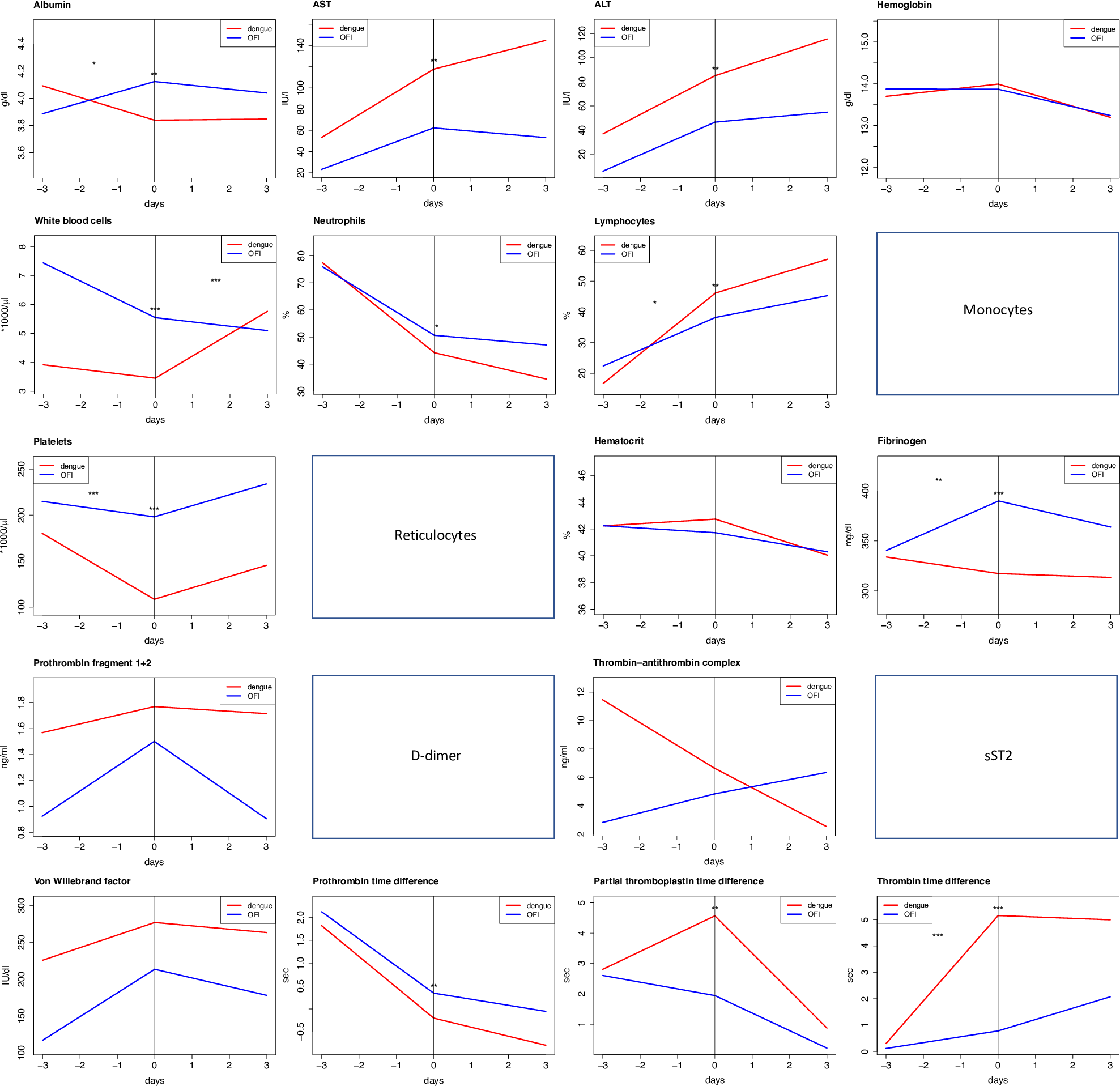

Supplement: S4 Fig — No results displayed for D-dimer, monocytes, reticulocytes and sST2 as the models could partially not be fitted. *p-value ≤ 0.05; **p-value ≤ 0.01; ***p-value ≤ 0.001. (TIF) [file pntd.0008199.s012.tif]

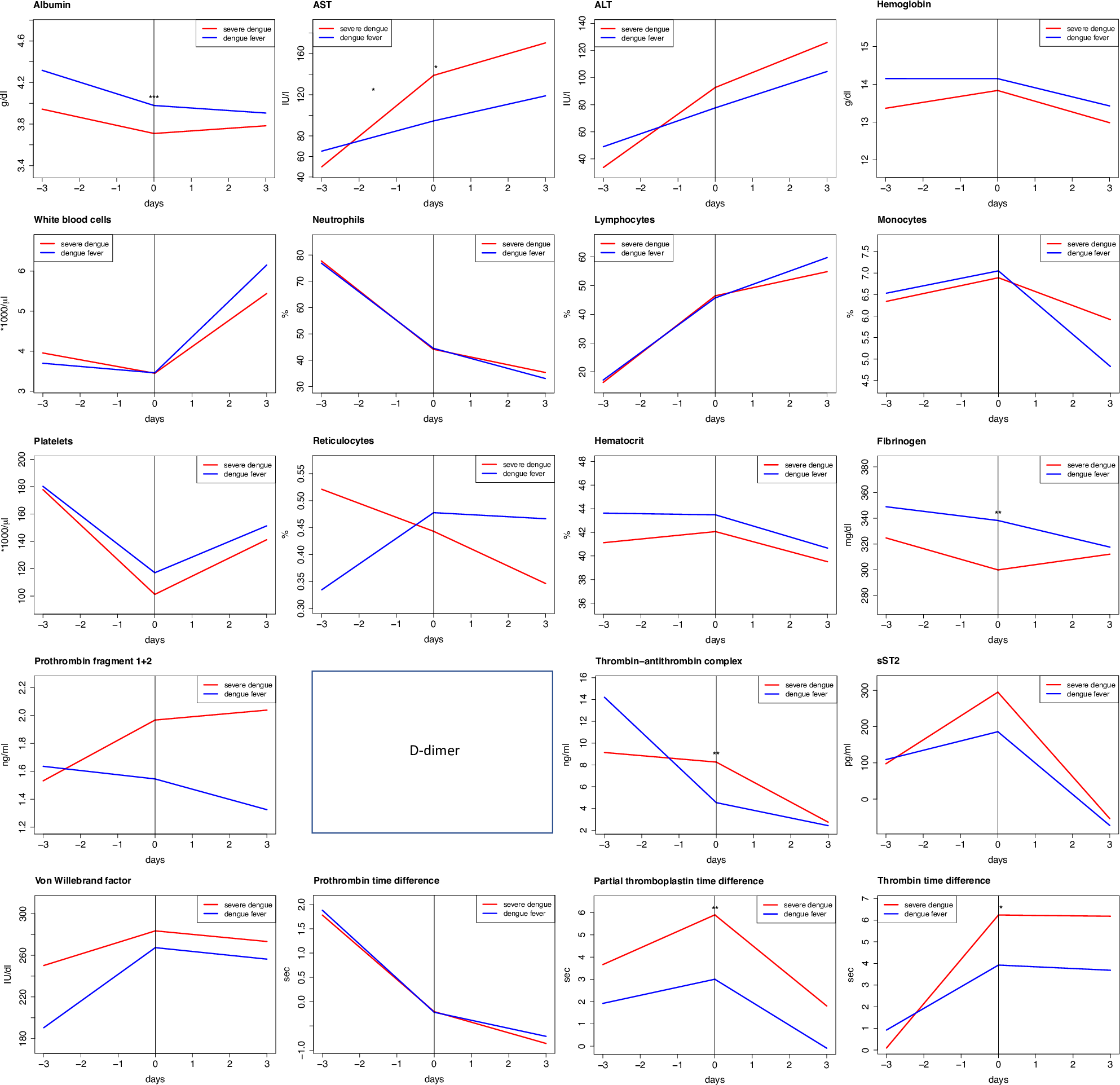

Supplement: S5 Fig — No results displayed for D-dimer as the models could partially not be fitted. *p-value ≤ 0.05; **p-value ≤ 0.01; ***p-value ≤ 0.001. (TIF) [file pntd.0008199.s013.tif]

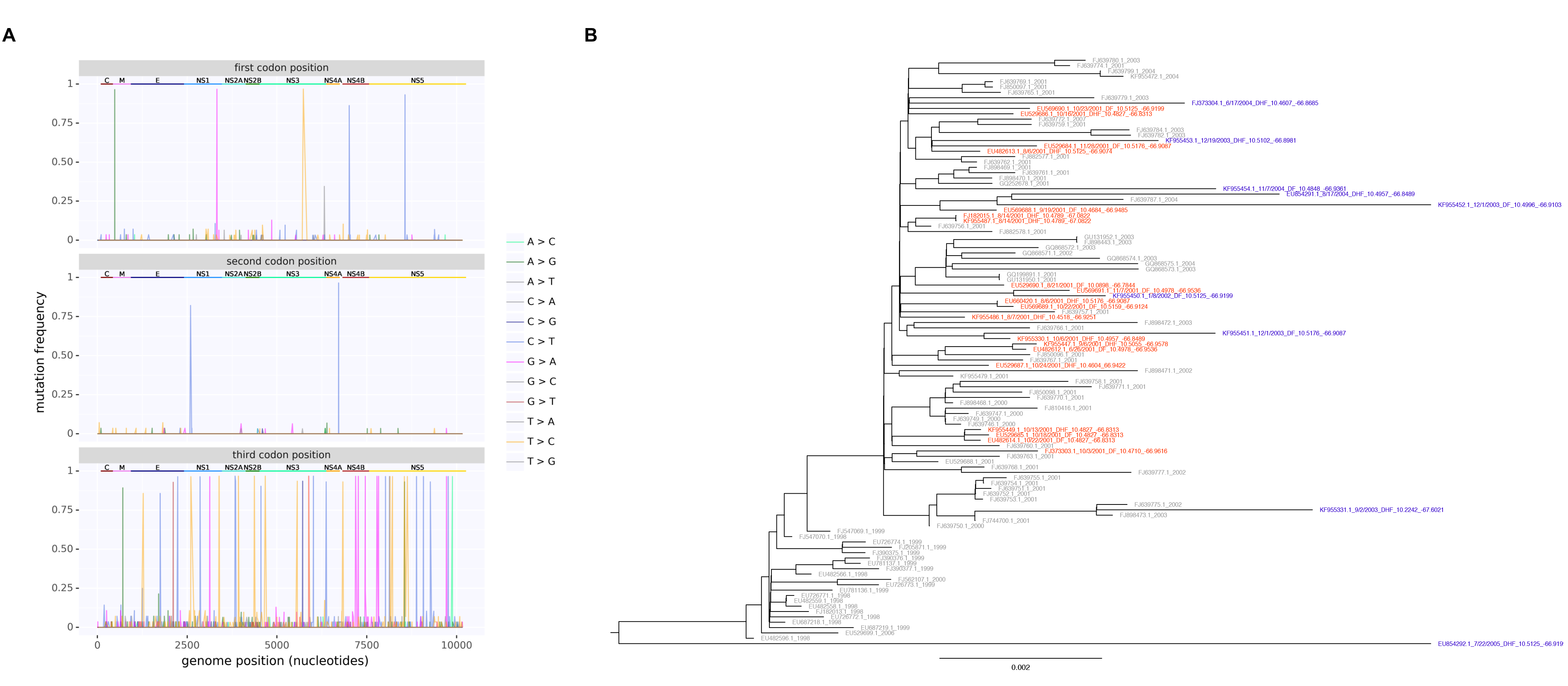

Supplement: S6 Fig — A. Distribution of amino acid mutations across the DENV-3 genome, separated by codon positions. B. Phylogenetic tree showing Caracas samples collected in 2001 in red, other Caracas samples in blue, and isolates from nearby locations in grey. Tree was found using RAxML rapid bootstrapping with 100 bootstrap replicates. (TIFF) [file pntd.0008199.s014.tiff]
