# Supplementary material for: Multivariate time-series analysis of biomarkers from a dengue cohort offers new approaches for diagnosis and prognosis
Source: PLoS Negl Trop Dis. 2020 Jun 16;14(6):e0008199. doi: 10.1371/journal.pntd.0008199 (PMC7380649; doi:10.1371/journal.pntd.0008199)
Supplement: Attachment — Submitted filename: Response to Reviewers_Caracas.pdf [file pntd.0008199.s016.pdf]

501 Massachusetts Avenue
Cambridge, MA 02139

February 2nd, 2020.

Abdallah M. Samy, PhD
Guest Editor
PLOS Neglected Tropical Diseases

Robert Reiner
Deputy Editor
PLOS Neglected Tropical Diseases

Re: PNTD-D-19-01624

Dear Editors,

We have addressed each of the points raised from the reviewers and the letter below contains point by point our comments and answers. In addition, the track changes in the new version of the manuscript are provided in a separate document to show the various modifications that were introduced as a consequence of the review process.
The Authors are very much in agreement with the various points raised and believe that after these iterations the manuscript is substantially better.
We thank the reviewers for the time and interests in carefully critiquing the present manuscript and we hope that the reviewers find the answers acceptable.

Sincerely yours,

Irene Bosch, PhD.

Response to Reviewers

Reviewer #2:

1-Human ethics number required?

ANSWER: *Yes, it is required. The number is IRB H-3693 (line 207). NIH grant required approval of the local (Caracas, Venezuela) and USA (Umass Medical School IRB ethical committee). There is one clinical study where all the samples were obtained therefore, there is only one IRB number listed in the manuscript*

2- Lines 237-238 and 511-514: 31 out of how many samples yielded suitable genome sequences?

ANSWER: *Only 31 DENV-3 genomes were sequenced; 43 samples were submitted for genome analysis.*

3-Some manufacturer's details are missing (e.g. Dengue IgM, IgG reagents, antibodies etc)

ANSWER: *We agree that some details were missing because the reagents were made and purchased by the National Institute of Health of Venezuela, they were a partner in the clinical study to execute serological tests. The technique we used for IgM ELISA was published elsewhere, it is based on the MAC ELISA from the Instituto Nacional de Salud in Venezuela (reference added), and IgG ELISA was commercially available (PanBio). We have added a reference in the Bibliography to appropriately address this issue (lines 251-253).*

4-Since most statistical results are provided, the authors could include exact p values rather than just general significance.

ANSWER: *We agree with the reviewer and modified all the figures according to the suggestion. The new version of the manuscript shows the corresponding p values in the revised Figure 3.*

Reviewer #3:

1-One IRB approval is listed but there are several affiliations of authors. Was IRB approval sought at other study sites?

ANSWER: *Since there is one clinical site where all samples and data were collected (Banco de Sangre, Caracas, Venezuela and one parent grant at University of Massachusetts Medical School, Worcester MA directly working with Banco de Sangre), there is one approved IRB that links those institutions. No further data collection was executed outside of IRB; rather, additional authors only used deidentified data approved under that IRB for their analysis. IRB number has been added to the manuscript (line 207).*

2- The visit schedule is not clear in the "Data collection" section of the methods

ANSWER: *Agree, the Data Collection has been added to the method section (lines 210-223). Also, we now show a schedule of the visits in Figure 1A.*

3- For the genome analysis, it is not clear what the mutations were in comparison to? Was a reference sequence used or was this observed temporally during the outbreak? Please deposit the sequences at the time of publication (not only supplemental but in a repository).

ANSWER. Agree with the reviewer, on lines 235-235 we added a the list of the gene bank accession numbers which have already been added to the repository at NCBI. A refence sequence was not used, rather a consensus sequence using the 31 genomes was generated and used as a reference.

Reviewer #2:

1-Whilst providing statistical significance e.g. by * p<0.01 is commonly performed, stating exact p values are more suited here (include in figures).

We provided the p values in figures as requested.

2-All figures should include in figure legend or better on y-axes what is depicted, e.g. mean ± 95% CI. Please include this info in all relevant figures. Supp files have often 'median' in the figure legend, which is acceptable if no ranges are given.

We provided the information as requested.

3-Table 1: age sign >=7 should be ≥;

We made the change as requested (line 841).

4-L290 states 61% were female, whereas Table 1 depicts 8 females in total in the study population. Please clarify.

We corrected the table accordingly (two rows had been interchanged in the new version of the manuscript) (line 841).

5-Table 2: plateletes = typo, correct to platelets.

We corrected the mistake, as requested (line 849).

6-Figure 1: include number of subjects in brackets under each criteria/condition. Change notes * to another symbol to avoid confusion with statistical significance symbol (*).

We provided the change as requested, new Fig. 1B.

7-Figure 4A needs a description of what total count is depicted: mean? Median? Please include.

We provided the description as requested (line 893).

8-Sup Fig 6A needs to be increased as not legible at 400% zoom

We changed the figure as requested.

9-Abbreviations: check throughout, e.g. use MCP-1, use vWF in figures if used abbreviation in text.

We reviewed these inconsistencies and made changes accordingly in the new version of the manuscript.

10- L149-153, why were not some other factors tested that are known in Dengue pathobiology? E.g. MCP-1, TNFa, IFy, etc? Include a comment in results or discussion. It would have been nice to confirm findings from other researchers.

ANSWER: The cytokines the reviewer suggested are present in dengue infection, but are not dengue specific. As such, when we performed the study, we used cytokines that are characteristic of dengue based on our previous published studies.

Reviewer #3:

1-The abstract makes a nice distinction between the time points where these factors were significantly increased/decreased (e.g. at defervescence) but this is not clear from the results section.

We provided the changes as requested. Added text in the results section as indicated in highlighted areas in the new version of the manuscript (lines 323-332, 340-349, 358-366).

2-Lines 312-13 Provide the time point? The abstract indicates at defervescence? Provide the data for sST2 that was not significant?

We provided the changes as requested (line 328). The data that was not significant is between DF and SD group Day -1 to Day +1 (lines 342-344).

3-Figure 2, can plots be provided or absolute values (potentially as a supplemental data set)? The heat map is not specific enough and is difficult to read. Also, the interpretation of differences between significance for the kinetic and intercept are not described adequately or provided context for the broader audience.

ANSWER: Agree with the reviewer. The authors would like to thank the reviewer for careful and critical comment of this figure. We provided the changes as requested. We re-did the heat map based on the numeric values and represented quantitative color scales instead.

4-Figure 3 legend does not indicate what is meant by Day 0. Fever? Recruitment? Defervescence? Were there other clinical parameters that were collected that were not significant?

We provided the changes as requested. Fever is meant as the day of defervescence (line 888-889). All other clinical parameters were included in supplemental figures.

5- For the clinical parameters provided in Fig. 2-3 can the AUC values be provided/discussed?

We provided the AUC values in the supplementary material.

Reviewer #1:
1-A shortcoming, which may be attributed to the nature of the study design, is the lack of a control group. Of the 204 patients in the study population, 55 were excluded because they did not present with fever and one or more typical symptom, febrile illness for less than two days and no other obvious infection. It would be interesting, for future studies to also follow the progression of individuals who are asymptomatic but test positive for the presence of dengue RNA. This would enhance the scope of the study to also elucidate which biomarkers are of importance in the symptomatic developments of dengue fever at early timepoints in which individuals are viremic but asymptomatic and their subsequent progression to severe dengue.

ANSWER: Due to the fact that this was a hospital prospective cohort it was difficult to recruit asymptomatic patients. The comment is well taken and we look forward to do other clinical studies in the near future to capture disease in the population rather than upon consultation at the medical centers. Nevertheless, we compared confirmed dengue patients to other febrile illnesses that were not due to dengue infection.

Reviewer #3:
1-The abstract does not make it clear whether the transitions observed in genomes are reported as a marker of severity or some other association.

We provided the changes as requested in the new version of the Abstract (lines 75-76).

2-Although the intro and discussion adequately discuss the potential of clinical measures to predict SD, there is no acknowledgment of the development of biomarkers for SD prediction.

We provided the changes as requested in the new version of the discussion section (lines 458-459).

3-Line 419- What do the authors consider a “hemostatic factor”. Also the use of the terms “hemodynamically stable” versus “unstable” are used throughout the discussion but how the authors define this is unclear.

Hemodynamically stable and unstable refers to a patient who has normal or abnormal blood pressure, heart rate, and abnormal capillary refill. We have reworded the discussion such that we no longer use the terms “hemostatic factor and hemodynamically stable/unstable.”

4-Line 463, this data is not included in the results and should be.

We refer the reader to Supplementary Fig. 6B (line 504), where the lower branch indicates earlier genomes from the Caribbean.

5-The title suggests that new approaches to diagnosis/prognosis are revealed by the data, but that is not fully clear since many of the variables used for this analysis have been considered by other groups and are either not consistent from cohort to cohort or no protocol exists for converting these values into a prognostic strategy.

The novelty of the study was further explained in the new version of the manuscript (lines 475-480, 481-489). While albumin, fibrinogen, lymphocytes, platelets, and TT have previously been published as dengue diagnostic/prognostic markers, our novel time-series analysis suggest that at least two consecutive days of consultation and laboratory tests increases diagnostic power. By normalizing data to the defervescence day (D0), clearly demonstrates the clinical parameters that are considered early versus late differentiators of diagnosis and prognosis.

As such, we would like propose to keep the original title.

Reviewer #3:

1-Specifically Figure 2 doesn't contain any raw data, only a heat map that was generated by the authors and not a statistical program regarding p-values. This needs to be improved and some representation of data itself included.

We provided the changes as requested for Figure 2.
