# Supplementary material for: Multivariate time-series analysis of biomarkers from a dengue cohort offers new approaches for diagnosis and prognosis
Source: PLoS Negl Trop Dis. 2020 Jun 16;14(6):e0008199. doi: 10.1371/journal.pntd.0008199 (PMC7380649; doi:10.1371/journal.pntd.0008199)
Supplement: Attachment — Submitted filename: PNTD-D-19-01624 Vasey et al review.docx [file pntd.0008199.s015.docx]

**Revision of Journal reference:** PNTD-D-19-01624

**Title**: Multivariate time-series analysis of biomarkers from a dengue cohort offers new approaches for diagnosis and prognosis

**General comments:** This manuscript authored by Dr Vasey *et al* discusses clinical and hematological parameters that could assist in distinguishing Dengue fever from other febrile illnesses. It has been an interesting read, considering the data/samples were acquired >14 years ago.

**Introduction**

- is in general well written.
- It lacks a summary of what is presented here and implications of the author’s main findings for the scientific community (guidelines state “The Introduction should conclude with a brief statement of the overall aim of the experiments and a comment about whether that aim was achieved.”)

**Methods**

- Are failry clear, concise.
- Nice description of the developed statistical analyses in R
- Human ethics number required?
- L 223: 31 out of how many samples yielded suitable genome sequences?
- Some manufacturer’s details are missing (e.g. Dengue IgM, IgG reagents, antibodies etc)
- Since most statistical results are provided, the authors could include exact p values rather than just general significance.

**Results/Figures/Tables**

- Whilst providing statistical significance e.g. by * p<0.01 is commonly performed, stating exact p values are more suited here (include in figures).
- All figures should include in figure legend or better on y-axes what is depicted, e.g. mean ± 95% CI. Please include this info in all relevant figures. Supp files have often ‘median’ in the figure legend, which is acceptable if no ranges are given.
- Table 1: age sign >=7 should be ≥;
- L290 states 61% were female, whereas Table 1 depicts 8 females in total in the study population. Please clarify.
- Table 2: plateletes = typo, correct to platelets.
- Figure 1: include number of subjects in brackets under each criteria/condition. Change notes * to another symbol to avoid confusion with statistical significance symbol (*).
- Figure 4A needs a description of what total count is depicted: mean? Median? Please include.
- Sup Fig 6A needs to be increased as not legible at 400% zoom
- Abbreviations: check throughout, e.g. use MCP-1, use vWF in figures if used abbreviation in text
- L149-153, why were not some other factors tested that are known in Dengue pathobiology? E.g. MCP-1, TNFa, IFy, etc? Include a comment in results or discussion. It would have been nice to confirm findings from other researchers.

**Discussion**

- Limitation: include other parameters already associated with DS/DSS
- Lacks the discussion of assessing the potential fever origin from other sources (e.g. other underlying infection)
- Final conclusion/summary should maybe contain the comment that changes in the measured factors (albumin, fibrinogen, D-dimer, thrombin-antithrombin complex, aPTT, thrombin changes etc) of SD on the day of effervescence are most likely linked to Dengue. But since no other tests were carried out assessing other underlying diseases/infections, this remains to be verified.

In summary, this manuscript is acceptable for publication upon completion of the suggested edits.
